# Establishment and effectiveness evaluation of a scoring system for exertional heat stroke by retrospective analysis

**DOI:** 10.1186/s40779-020-00269-1

**Published:** 2020-08-27

**Authors:** Meng-Meng Yang, Lu Wang, Yu Zhang, Rui Yuan, Yan Zhao, Jie Hu, Fei-Hu Zhou, Hong-Jun Kang

**Affiliations:** 1 Department of Critical Care Medicine, The First Medical Centre, Chinese PLA General Hospital, No. 28, Fuxing Road, Haidian District, Beijing, 100853 China; 2 Medical School of Chinese PLA, Beijing, China

**Keywords:** Exertional heat stroke, Scoring system, Prognosis, Effectiveness of evaluation

## Abstract

**Background:**

Heat stroke (HS) is a serious, life-threatening disease. However, there is no scoring system for HS so far. This research is to establish a scoring system that can quantitatively assess the severity of exertional heat stroke (EHS).

**Methods:**

Data were collected from a total of 170 exertional heat stroke (EHS) patients between 2005 and 2016 from 52 hospitals in China. Univariate statistical methods and comparison of the area under the receiver operating characteristic (ROC) curve (AUC) were used to screen exertional heat stroke score (EHSS) parameters, including but not limited body temperature (T), Glasgow Coma Scale (GCS) and others. By comparing the sizes of the AUCs of the APACHE II, SOFA and EHSS assessments, the effectiveness of EHSS in evaluating the prognosis of EHS patients was verified.

**Results:**

Through screening with a series of methods, as described above, the present study determined 12 parameters – body temperature (T), GCS, pH, lactate (Lac), platelet count (PLT), prothrombin time (PT), fibrinogen (Fib), troponin I (TnI), aspartate aminotransferase (AST), total bilirubin (TBIL), creatinine (Cr) and acute gastrointestinal injury (AGI) classification – as EHSS parameters. It is a 0–47 point system designed to reflect increasing severity of heat stroke. Low (EHSS< 20) and high scores (EHSS> 35) showed 100% survival and 100% mortality, respectively. We found that AUCEHSS > AUCSOFA > AUCAPACHE II.

**Conclusion:**

A total of 12 parameters – T, GCS, pH, Lac, PLT, PT, Fib, TnI, AST, TBIL, Cr and gastrointestinal AGI classification – are the EHSS parameters with the best effectiveness in evaluating the prognosis of EHS patients. As EHSS score increases, the mortality rate of EHS patients gradually increases.

## Background

Heat stroke (HS) is a serious, life-threatening disease characterized by elevated core body temperature that is simultaneously accompanied by central nervous system (CNS) dysfunction [1]. The disease is often accompanied by multiple-organ dysfunction [2, 3]. HS is mainly divided into two types: classical heat stroke (CHS) and exertional heat stroke (EHS). The former more frequently occurs in children and the elderly, who are exposed to high temperatures, while the latter mostly occurs in healthy young populations who are engaged in high-intensity manual labor, including soldiers, athletes, workers and farmers [4]. Compared with CHS, each organ injury of EHS is more serious [1]. EHS has become the third leading cause of death among athletes [5].

As a criterion for evaluating the severity of disease in intensive care units (ICUs), scoring systems have become important tools to help clinical physicians to make decisions [6]. At present, scoring systems for severe diseases are mainly divided into two major types. One type of scoring system is suitable for various diseases, such as the Acute Physiology and Chronic Health Evaluation II (APACHE II), the Simplified Acute Physiology Score (SAPS II) and the Multiple Organ Dysfunction Score (MODS); the other type of scoring system is for a specific type of organ or disease, such as the Glasgow Coma Scale (GCS) for evaluating the degree of CNS injury and the Ranson scale for evaluating the degree of injury of severe acute pancreatitis [7, 8]. At present, the scoring systems that are used to evaluate the severity of EHS are mainly APACHE II and SOFA [9, 10]. Studies have found that the common causes that affect the prognosis of EHS are disseminated intravascular coagulation (DIC) caused by coagulation dysfunction and rhabdomyolysis induced by thermal injury [11, 12]. The above-mentioned scoring systems ignore the exact roles played by the above two indicators in the scoring system, with the result that none of the existing scoring systems can evaluate the condition of EHS very objectively and comprehensively, including the systemic scores of APACHE II, SOFA, SAPSII and MODS [13–16]. As early as 12 years ago, Varghese et al. [17] suggested that a scoring system dedicated to HS should be established to stratify disease severity and prognosis, which is very important for choosing optimal treatment strategies and improving the success rates of clinical treatment. Once an exertional heat stroke score (EHSS) is finalized, critical care can be accessed sooner, and it may be used in the future to stratify patients for heat stroke-specific treatment. However, more than a decade has passed, and an effective HS scoring system has not yet been established. Similarly, two well-known specialists in the HS field, Professor Leon and Bouchama [1], both noted that APACHE II scoring was not a specific scoring criterion for HS. EHS has a characteristic dispersed outbreak, which makes it very difficult for researchers to perform large sample size-based clinical studies [18]. This limitation may be the underlying reason that prevents the EHS scoring system from being established.

In summary, creating a scoring system that is consistent with the pathological and physiological characteristics of EHS has clinical significance in evaluating disease, judging treatment efficacy and determining prognosis. The main purpose of this study is to screen and identify the parameters that are consistent with the pathological and physiological characteristics of EHS through a retrospective study to assign corresponding values, to create a scoring system for exertional heat stroke – the EHS score (EHSS) – and to confirm its effectiveness at evaluating EHS. The mortality rates of EHS patients corresponding to different EHSS scores were also investigated.

## Methods

### Setting

We collected data from a total of 170 EHS patients between 2005 and 2016 from 52 hospitals in China, including the People’s Liberation Army (PLA) General Hospital. Ninety patients were randomly selected as the study subjects for establishing the EHSS, of which 69 patients (76.7%) survived, and 21 (23.3%) died. The remaining 80 patients were selected as the study subjects to verify the effectiveness of the EHSS in evaluating the prognosis of EHS patients. Of these, 62 (77.5%) survived and 18 (22.5%) died. The sources of the patients were widely distributed across various provinces of China except Tibet, and the geographic distribution of the cases was uniform. The study was led by the PLA General Hospital and was approved by the ethics committees of all 52 hospitals.

### Patients and study design

In this study, the EHS patient population mainly included soldiers, athletes, workers and farmers. Inclusion criteria were as follows: 1) patients whose age was no less than 18 years; 2) patients with a history of being engaged in high-intensity manual labor; 3) patients whose axillary temperature was higher than 39 °C (studies find that the rectal temperature, which represents the core body temperature of humans, is usually higher than the oral temperature by 0.27 °C–0.38 °C, whereas the oral temperature is 0.55 °C higher than the axillary temperature, which represents body surface temperature) [19]; and 4) patients with CNS dysfunction, including delirium, coma, disturbances of consciousness and disorientation. The included patients met all of the above four criteria. The exclusion criteria were as follows: 1) patients who were in the hospital or ICU for less than 24 h; and 2) patients with common comorbidities before EHS onset; in our research, they had diabetes, cerebral infarction, pulmonary infection and dementia. Gastrointestinal tract injury was graded by the acute gastrointestinal injury (AGI) classification developed by the European Society of Intensive Care Medicine (ESICM) in 2012 [20].

### Screening of the scoring system parameters for exertional heat stroke

According to our clinical experience, a literature review and the consensus of heat stroke specialists [21], the present study first screened 42 physiological parameters that could reflect the severity of the pathological changes of various systems in the body, particularly indicators that are characterized by high fever, rhabdomyolysis, coagulation dysfunction and nervous system dysfunction, as shown in Table 1. The worst values of the physiological parameters within 24 h after admission into the hospital or ICU were selected. First, univariate statistical analysis was used to preliminarily screen EHSS parameters. When the differences were statistically significant, their areas under the receiver operating characteristic (ROC) curves (AUCs) were compared. The diagnostic accuracy for diseases is low when the AUC is 0.5–0.7, acceptable when the AUC is 0.7–0.9 and high when the AUC is above 0.9; therefore, the present study selected the parameters with AUCs> 0.7 as the final parameters for EHSS. By combining our clinical experience and a literature review, the final parameters for EHSS were determined. Multivariate logistic regression analysis is a statistical method that requires data from a large sample size; the smaller the sample size is, the less reliable the results will be [22]. At present, there is still no well-accepted effective sample size calculation formula for multivariate logistic regression analysis; the well-accepted sample size by academics is at least 10 times the number of parameters that are included in the multivariate logistic regression analysis. In the present study, a total of 23 parameters needed to be included in the multivariate logistic regression equation; therefore, if logistic regression analysis was to be performed, the number of EHS patients needed to be at least 230 each for the death and survival groups, for a total of 460 cases, to ensure reliable results. However, the EHS patients for this part of the study numbered only 90. Therefore, logistic regression analysis was not suitable for the screening of EHSS parameters. In contrast with multivariate logistic regression, the ROC curve is a statistical method that can be used for data analysis with a small sample size. Therefore, in the present study, we calculated the AUCs of the 23 parameters whose differences in univariate analysis were statistically significant, and we identified the parameters with AUCs greater than 0.7 as the final EHSS parameters.

**Table 1:** The screening parameters for EHSS

Classification	Parameters					
Vital signs	T	MAP	RR	HR		
Central nervous system	GCS					
Blood gas analysis	PH	SaO_2_	HCO_3_^−^	Lac		
Routine blood test	Hb	WBC	N%	PLT		
Cardiac function	BNP	CK-MB	TnI	LDH		
Hepatic function	ALT	AST	TBIL	DBIL	ALB	
Kidney function	Cr	BUN				
Rhabdomyolysis	Mb	uMb	CK			
Coagulation	PT	APTT	TT	Fib	D-D	FDP
Inflammation markers	CRP	IL-6	PCT			
Electrolyte	K^+^	Na^+^	Cl^−^	Ca^2+^		
Metabolic parameters	Glu					
Gastrointestinal function	AGI(I—IV)					

*T* Temperature, *MAP* Mean arterial pressure, *RR* Respiratory rate, *HR* Heart rate, *GCS* Glasgow Coma Scale, *SaO*_*2*_ Oxygen saturation, *Lac* Lactate, *Hb* Hemoglobin, *WBC* White blood cell count, *N* Neutrophils, *PLT* Platelets, *BNP* Brain natriuretic peptide, *CK-MB* Creatine kinase isoenzymes, *TnI* Troponin I, *LDH* Lactate dehydrogenase, *ALT* Alanine transaminase, *AST* Aspartate transaminase, *TBIL* Total bilirubin, *DBIL* Direct bilirubin, *ALB* Albumin, *Cr* Creatinine, *BUN* Blood urea nitrogen, *Mb* Myoglobin, *uMb* uric myoglobin, *CK* Creatine kinase, *PT* Prothrombin time, *APTT* Activated part of the Prothrombin Time, *TT* Thrombin time, *Fib* Fibrinogen, *D-D* D-dimer, *FDP* Fibrin degradation products, *CRP* C-reactive protein, *IL-6* Interleukin-6, *PCT* Procalcitonin, *K*^*+*^ Potassium, *Na*^*+*^ Sodium, *Cl*^−^. Chlorine, *Ca*^*2+*^ Calcium, *Glu* Glucose, *AGI* Acute gastrointestinal injury

### Establishment of a scoring system for exertional heat stroke

The method of value assignment to parameters of the MODS scoring system was used as a reference. Values were assigned to parameters according to the mortality rates corresponding to different variable ranges of various parameters. When the variable range was given 4 points, its corresponding ICU mortality rate should be greater than 50% [16]. The assignment of values to parameters gave corresponding grade points to physiological variables with the value assignment methods of the APACHE II and MODS scoring systems as the references, and the assignment was divided into five grades according to the abnormalities of the parameters, with various variables having assigned values of 0–4 points [13, 16].

Verification of the evaluation effectiveness of EHSS on the prognosis of EHS patients.

The worst values of the APACHE II, SOFA and EHSS scoring systems within 24 h after admission to the hospital or ICU were calculated for the 80 EHS patients. The AUCs of the three scoring systems were calculated, and the predictive effectiveness of the EHSS on EHS patients was judged by comparing the sizes of the AUCs of the three scoring systems.

### Exploring the correlations between different EHSS scores and the prognosis of EHS patients

The EHSS score of each EHS patient was calculated. The scores were grouped into 5-point intervals, and the total mortality rates of EHS patients corresponding to various EHSS score intervals were calculated.

### Statistical analysis

First, the EHS patients were divided into death and survival groups according to prognosis. Measurement datasets with normal distribution are represented by means ± standard deviations (*x* ± *s*), while datasets without normal distribution are represented by medians (interquartile ranges). Count data are expressed as percentages. For the comparison of data between two groups, the univariate statistical method was used for measurement data, the two-independent-samples t-test was used for two sets of data with a normal distribution, and the rank sum test for two independent samples was used for data without a normal distribution. The χ^2^ test was used for count data. SPSS 17.0 was used for statistical analysis of the above types. Several parameters for some patients were not detected within 24 h after admission into the hospital or ICU, such as HCO^3−^, TnI, D-D, Cl^−^ and Ca^2+^. Because these parameters had missing data during data collection, the mean imputation method for treating the missing data would, to a certain extent, affect the authenticity of the result; therefore, in the present study, we discarded the missing data and calculated the missing rates of various parameters. For the parameters in which the difference in the univariate analysis was statistically significant, the ROC curve was used, the AUC was calculated to screen EHSS parameters, and the 95% confidence interval was calculated. The AUCEHSS meant the area under the ROC curve of EHSS, AUCAPACHE II meant the area under the ROC curve of APACHE II, and AUCSOFA meant the area under the ROC curve of SOFA. MedCalc 15.8 was used for statistical analysis of the data. In this study, *P* < 0.05 was considered statistically significant.

## Results

### Demographic characteristics and baseline clinical data

The demographics and baseline clinical data of the EHS patients for EHSS establishment and EHSS verification are shown in Table 2. The cooling effect and core body temperature dropped to 38.5 °C within 2 h after EHS onset. The APACHE II score of the nonsurvival group was significantly higher than that of the survival group, and the proportion of patients in the nonsurvival group who used vasoactive agents within 24 h after admission to the hospital or ICU was significantly higher than that of the survival group, suggesting that the condition of EHS patients in the death group within 24 h of admission to the hospital or ICU was more serious than that of the patients in the survival group. When comparing the baseline level of EHS patients for establishing EHSS to the baseline level of EHS patients for verifying the evaluation effectiveness of EHSS, there were no significant differences in various categories. Patients in the two categories had good homogeneity (as shown in Table 3).

**Table 2:** The demographics and baseline clinical data of the EHS patients for EHSS establishment and EHSS verification

Variables	Total	Survival group	Nonsurvival group	*P*
EHSS establishment (*n*)	90	69	21	
Male [*n*(%)]	84 (93.3)	64 (92.8)	20 (95.2)	1.000
Age [year, M(Q)]	22 (19–32)	22 (19–26.5)	27 (20.5–37.5)	0.066
APACHE II score (*x* ± *s*)	23.5 ± 8.6	20.7 ± 7.4	32.8 ± 4.7	0.048
Vasoactive drugs [*n*(%)]	25 (27.8)	14 (20.3)	11 (52.4)	0.013
Cooling effect [*n*(%)]	32 (35.6)	27 (39.1)	5 (23.8)	0.161
EHSS verification (*n*)	80	62	18	
Male [*n*(%)]	72 (90.0)	58 (93.5)	14 (77.8)	0.071
Age [year, M(Q)]	24 (20–30)	25 (20.7–30.2)	20.5 (19.7–27.2)	0.146
APACHE II score (*x* ± *s*)	21.2 ± 7.3	18.9 ± 5.9	29.1 ± 6.4	< 0.001
Vasoactive drugs [*n*(%)]	29 (36.3)	17 (27.4)	12 (66.7)	0.002
Cooling effect [*n*(%)]	35 (43.8)	29 (46.8)	6 (33.3)	0.312

*EHSS* Exertional Heat Stroke Score, *APACHE II* Acute Physiology and Chronic Health Evaluation II score, Cooling effect. Core body temperature dropped to 38.5 °C within 2 h after EHS onset

**Table 3:** Comparison of demographics and baseline clinical data between the EHSS establishment group and the EHSS verification group

Variables	Total (*n* = 170)	EHSS establishment group (*n* = 90)	EHSS verification group (*n* = 80)	*P*
Male/Female	156/14	84/6	72/8	0.430
Age [year, M(Q)]	22 (20–31)	22 (19–32)	24 (20–30)	0.598
APACHE II score (*x* ± *s*)	22.4 ± 8.1	23.5 ± 8.6	21.2 ± 7.3	0.066
Vasoactive drugs [*n*(%)]	54 (31.8)	25 (27.8)	29 (36.3)	0.236
Cooling effect [*n*(%)]	67 (39.4)	32 (35.5)	35 (43.8)	0.275
Death [*n*(%)]	39 (22.9)	21 (23.3)	18 (22.5)	0.897

EHSS. Exertional Heat Stroke Score; APACHE II. Acute Physiology and Chronic Health Evaluation II score; Cooling effect. Core body temperature dropped to 38.5 °C within 2 h after EHS onset

### Results of screening the scoring system parameters for exertional heat stroke

The univariate statistical analysis of identified EHSS parameters that were actually included in the statistical analysis is shown in Table 4. A total of 23 parameters with significant differences between the two groups were included in the EHSS for the next round of screening.

**Table 4:** Univariate statistical analysis for screening EHSS parameters

Parameters	Total (*n* = 90)	Survival group (*n* = 69)	Nonsurvival group (*n* = 21)	*P*
T [°C,M(Q)]	40.7 (40.0–41.2)	40.5 (39.4–41.0)	41 (40.5–42.0)	< 0.0001
MAP (mmHg, *x* ± *s*)	76.2 ± 19.1	75.7 ± 17.5	77.8 ± 23.9	0.06
RR [beats/min, M(Q)]	26 (20–30)	25 (20–30)	30 (26–35)	0.012
HR (beats/min, *x* ± *s*)	123 ± 35	118 ± 36	138 ± 27	0.089
GCS [score, M(Q)]	5.0 (3.0–8.0)	7.0 (3.5–9.0)	3.0 (3.0–3.5)	< 0.0001
pH [M(Q)]	7.37 (7.27–7.39)	7.37 (7.35–7.40)	7.23 (7.23–7.27)	< 0.0001
SaO_2_ [M(Q)]	92 (91–98)	92 (92–99)	91 (88–92)	0.007
HCO_3_^−^(mmol/L, *x* ± *s*, *n*^a^)	18.2 ± 4.8(*n*^a^ = 28)	19.5 ± 4.7(*n*^a^ = 24)	14.8 ± 3.4(*n*^a^ = 4)	0.429
Lac [mmol/L, M(Q)]	4.4 (3.6–7.3)	4.4 (3.3–4.8)	9.5 (8.0–10.5)	< 0.0001
Hb [g/L, M(Q)]	130.0 (118.0–138.0)	130.0 (121.5–138.5)	118.0 (99.5–139.0)	0.058
WBC (× 10^12^/L, *x* ± *s*)	14.4 ± 6.2	13.9 ± 5.4	16.0 ± 8.3	0.026
N [%, M(Q)]	89.5 (84.9–92.0)	89.1 (85.0–91.3)	90.3 (82.0–93.3)	0.44
PLT [× 10^9^/L, M(Q)]	55.0 (27.5–96.0)	64.0 (41.0–113.5)	24.0 (20.0–31.5)	< 0.0001
TnI [ng/ml, M(Q), *n*^b^]	0.25 (0.10–1.10)(*n*^b^ = 28)	0.19 (0.08–0.52) (*n*^b^ = 21)	2.30 (0.73–7.00)(*n*^b^ = 7)	< 0.0001
LDH [U/L, M(Q)]	942.0 (459.8–1736.5)	806.0 (393.5–942)	2397.0 (1375.0–2397.0)	< 0.0001
ALT [U/L, M(Q)]	231 (72.3–1087.8)	125 (64–456.5)	2995 (426.5–4296)	< 0.0001
AST [U/L, M(Q)]	379.5 (122.2–1354.2)	270 (94.5–685)	3533 (920–3860.5)	< 0.0001
TBIL [μmol/L, M(Q)]	33.1 (21.7–61.5)	28 (20.2–42)	107.7 (60.9–146.2)	< 0.0001
DBIL [μmol/L, M(Q)]	16 (9.4–28.9)	12.5 (8.7–19.3)	50.2 (32.4–52)	< 0.0001
ALB [g/L, M(Q)]	37.6 (35.2–40.9)	37.6 (35.1–41.3)	36.9 (35–40.7)	0.503
Cr [μmol/L, M(Q)]	145.5 (94.8–196.8)	136 (88.1–175.9)	237 (116.4–440.4)	0.001
BUN [mmol/L, M(Q)]	8.1 (6.3–9.9)	8.1 (6.1–9.6)	8.8 (6.5–16.2)	0.037
CK [U/L, M(Q)]	3412 (1588.3–6140.5)	2852 (1441.6–5723)	8850 (2327.7–35,237)	0.008
TT [s, M(Q)]	28.9 (18.7–37.8)	23.8 (17.9–37.8)	51 (29–56.7)	0.002
PT [s, M(Q)]	22.8 (16.4–43.2)	19.2 (15.8–26.5)	63.2 (39.6–93.2)	< 0.0001
APTT [s, M(Q)]	58.2 (37.4–96.7)	50.1 (34.8–75.8)	96.7 (61.1–125.9)	< 0.0001
Fib [g/L, M(Q)]	1.7 (1.12–2.04)	1.80 (1.48–2.13)	1.07 (0.59–1.42)	< 0.0001
D-D [μg/ml, M(Q), *n*^c^]	3.16 (1.09–6.65)(*n*^c^ = 30)	2.89 (1.07–8.52)(*n*^c^ = 22)	4.45 (1.76–5.93)(*n*^c^ = 8)	0.435
K^+^ [mmol/L, M(Q)]	3.4 (3.1–3.6)	3.4 (3.1–3.5)	3.6 (3.1–3.6)	0.204
Na^+^ [mmol/L, M(Q)]	137.8 (134.4–140.3)	137.8 (134.8–140.5)	135.9 (133.1–139.2)	0.112
Cl^−^ [mmol/L, M(Q) *n*^d^]	103.5 (99.7–106)(*n*^d^ = 26)	104 (101–106)(*n*^d^ = 20)	100.1 (99.0–104.8)(*n*^d^ = 6)	0.27
Ca^2+^ [mmol/L, M(Q), *n*^e^]	1.83 (1.12–2.04)(*n*^e^ = 20)	1.91 (1.16–2.09)(*n*^e^ = 15)	1.30 (1.04–1.75)(*n*^e^ = 5)	0.012
Glu [mmol/L, M(Q)]	8.57 (5.98–9.36)	8.77 (6.05–9.59)	7.57 (3.45–8.14)	0.055
AGI [n]				< 0.0001
I^a,b,c^	41	39	2	
II^d,e^	37	25	12	
III^f^	8	3	5	
IV	4	2	2	

*n*^a^ represents the missing values of HCO_3_^−^; the missing rates between the above two groups is *P* = 0.281; *n*^b^ represents the missing values of TnI; the missing rates between the above two groups is *p* = 0.802; *n*^c^ represents the missing values of D-D; the missing rates between the above two groups is *P* = 0.597; *n*^d^ represents the missing values of Cl^−^; the missing rates between the above two groups is *P* = 0.971; *n*^e^ represents the missing values of Ca^2+^, the missing rates between the above two groups is *P* = 0.842; ^a^Grade I compared with grade II, *P* = 0.002; ^b^Grade I compared with grade III, *P* = 0.001; ^c^Grade I compared with grade IV, *P* = 0.034; ^d^Grade II compared with grade III, *P* = 0.226; ^e^Grade II compared with grade IV, *P* = 0.596; ^f^ Grade III compared with grade IV, *P* = 1. *T* Temperature, *MAP* Mean Arterial Pressure, *RR* Respiratory Rate, *HR* Heart Rate, *GCS* Glasgow Coma Scale, *SaO2* Oxygen Saturation, *Lac* Lactate, *Hb* Haemoglobin, *WBC* White Blood Cell count, *N* Neutrophils, *PLT* Platelets, *TnI* Troponin I, *LDH* Lactate dehydrogenase, *ALT* Alanine Transaminase, *AST* Aspartate Transaminase, *TBIL* Total Bilirubin, *DBIL* Direct Bilirubin, *ALB* Albumin, *Cr* Creatinine, *BUN* Blood Urea Nitrogen, *CK* Creatine Kinase, *TT* Thrombin Time, *PT* Prothrombin Time, *APTT* Activated part of the Prothrombin Time, *Fib* Fibrinogen, *D-D* D-dimer, *FDP* Fibrin degradation products, *K+* Potassium, *Na+* Sodium, *Cl-* Chlorine, *Ca2+* Calcium, *Glu* Glucose, *AGI* Acute Gastrointestinal Injury

**Fig. 1:**
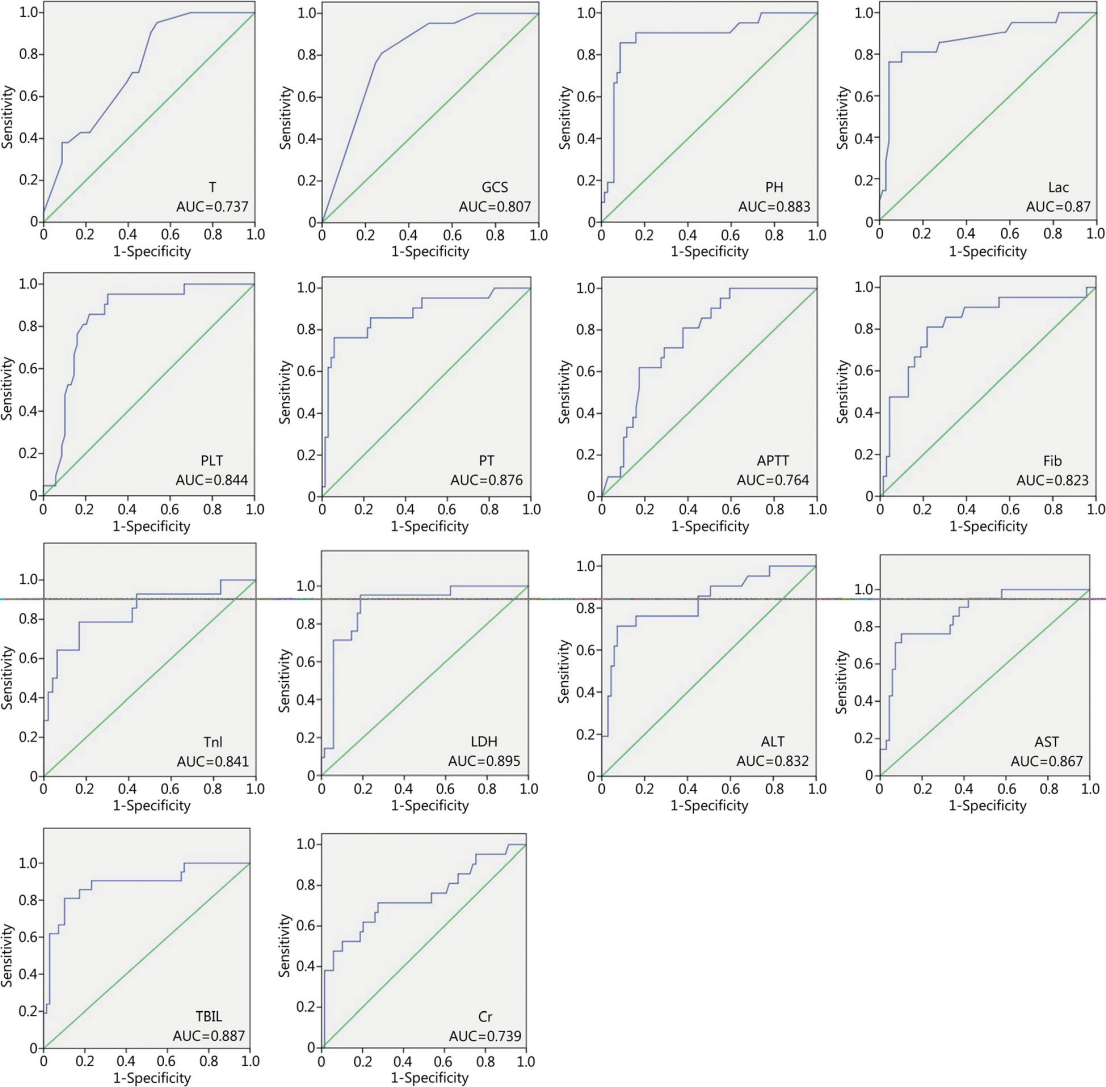
The AUCs of various parameters for EHSS

When the parameters with AUCs > 0.7 were taken as EHSS parameters, 14 were identified as EHSS parameters, as shown in Table 5. The AUCs of various parameters are shown in Fig. 1. When reviewing the establishment process of the classical scoring systems of APACHE II, sequential organ failure assessment (SOFA), MODS and SAPS II for critical and severe diseases, their included parameters were the most representative parameters capable of revealing injuries to various organs and systems [13–16]. When investigating the correlation between early coagulation function indicators and the prognosis of HS patients, previous clinical studies found that within 24 h of a patient’s admission to the hospital, the capability of predicting their prognosis by the three indicators platelet count (PLT), prothrombin time (PT) and activated partial thromboplastin time (APTT) decreased one by one [23], in contrast to this study. One clinical study on EHS showed that at the time the EHS patients were admitted to the hospital, 77% (24/31) showed increases in aspartate aminotransferase (AST), whereas the proportion of patients with increases in alanine aminotransferase (ALT) was only 39% (12/31) [18]. In the present study, AUCAST > AUCALT, suggesting that during early EHS, the diagnosis of acute liver injury by AST yielded a better result than diagnosis by ALT. Because lactate dehydrogenase (LDH) is widely distributed in various tissues, it has poor specificity in diagnosing myocardial injury. In contrast, troponin I (TnI) is a specific indicator to reflect myocardial injury, and its time to reach the peak value within 24 h is earlier than that of troponin T (TnT) [24]. Therefore, in this study, TnI was chosen as the indicator for myocardial injury. As seen from the statistical results in Table 5, according to AGI classification, in this study, there was a difference in gastrointestinal tract injuries between the two groups of EHS patients within 24 h after being admitted to the hospital or ICU. Through the series of screenings mentioned above, the present study eventually determined that the 12 parameters body temperature (T), GCS, pH, lactate (Lac), PLT, PT, fibrinogen (Fib), TnI, AST, total bilirubin (TBIL), creatinine (Cr) and gastrointestinal tract AGI could be classified as the parameters for EHSS.

**Table 5:** The screened parameters for EHSS by AUC

Parameters	AUC (95%CI)	Sensitivity (%)	Specificity (%)	Optimal cut-off value	*P*
T [°C, M(Q)]	0.737 (0.629–0.846)	95.2	46.4	40.1	< 0.0001
GCS [score, M(Q)]	0.807 (0.714–0.901)	72.5	81.0	4.5	< 0.0001
PH [M(Q)]	0.883 (0.791–0.974)	91.3	85.7	7.28	< 0.0001
Lac [mmol/L, M(Q)]	0.87 (0.771–0.97)	76.2	95.7	8.75	< 0.0001
PLT [×10^9^/L, M(Q)]	0.844 (0.757–0.93)	69.6	95.2	47.5	< 0.0001
PT [s, M(Q)]	0.876 (0.781–0.971)	76.2	94.2	47.1	< 0.0001
APTT [s, M(Q)]	0.764 (0.663–0.866)	61.9	82.6	88.4	< 0.0001
Fib [g/L, M(Q)]	0.823 (0.716–0.931)	78.3	81.0	1.43	< 0.0001
TnI [ng/ml, M(Q)]	0.841 (0.709–0.973)	78.6	83.3	0.785	< 0.0001
LDH [U/L, M(Q)]	0.895 (0.821–0.0.969)	95.2	81.2	1049	< 0.0001
ALT [U/L, M(Q)]	0.832 (0.723–0.942)	71.4	92.8	1407.5	< 0.0001
AST [U/L, M(Q)]	0.867 (0.782–0.951)	76.2	89.9	1312.0	< 0.0001
TBIL [μmol/L, M(Q)]	0.887 (0.797–0.976)	81.0	89.9	60.15	< 0.0001
Cr [μmol/L, M(Q)]	0.739 (0.604–0.875)	71.4	72.5	170	< 0.0001

*EHSS* Exertional Heat Stroke Score, *AUC* area under the receiver operating characteristic curve, *T* Temperature, *GCS* Glasgow Coma Scale, *Lac* Lactate, *PLT* Platelets, *PT* Prothrombin Time, *APTT* Activated part of the Prothrombin Time, *Fib* Fibrinogen, *TnI* Troponin I, *LDH* Lactate dehydrogenase, *ALT* Alanine Transaminase, *AST* Aspartate Transaminase, *TBIL* Total Bilirubin, *Cr* Creatinine

### Establishment of the scoring system for exertional heat stroke

**Fig. 2:**
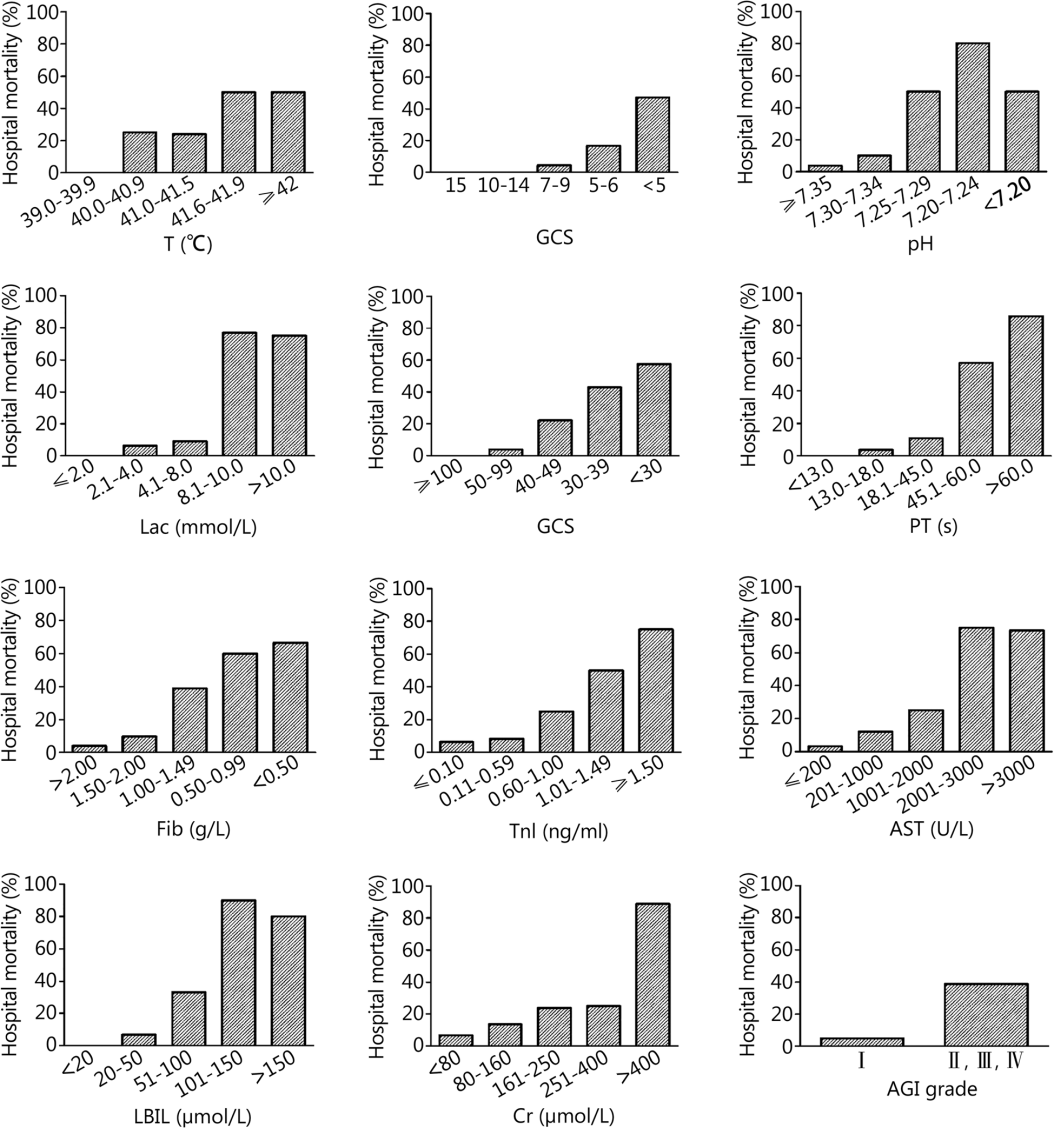
Mortality at different intervals of EHSS parameters

The mortality rates corresponding to different values of various parameters are shown in Fig. 2. As can be noted from the statistical results in Table 4, according to AGI classification, among EHS patients, the difference in the number of patients with grade I gastrointestinal injuries and the numbers of patients with grades II, III and IV gastrointestinal injuries was statistically significant between the two groups, whereas the difference in the numbers of patients with grades II and III gastrointestinal injuries, the numbers of patients with grades II and IV gastrointestinal injuries and the numbers of patients with grades III and IV gastrointestinal injuries between the two groups were not statistically significant. This result means that the effect of grade I gastrointestinal injuries are different from the effects of grades II, III and IV gastrointestinal injuries on the prognosis of EHS patients, but the difference between grades II, III and IV gastrointestinal injuries seems nonexistent. Therefore, in the present study, AGI grade I was assigned one point, and AGI grades II, III and IV were assigned 3 points. The detailed scoring results are shown in Table 6. The highest score of this scoring system was 47 points. At this point, EHSS establishment was completed.

**Table 6:** Detailed contents of Exertional Heat Stroke Score (EHSS)

Parameters	0 point	1 point	2 points	3 points	4 points
T(°C)	39–39.9	40–40.9	41–41.5	41.6–41.9	≥42
GCS	15	10–14	7–9	5–6	< 5
pH	≥7.35	7.30–7.34	7.25–7.29	7.20–7.24	< 7.20
Lac (mmol/L)	≤2.0	2.1–4.0	4.1–8.0	8.1–10.0	> 10
PLT (×10^9^/L)	≥100	50–99	40–49	30–39	< 30
PT (s)	< 13	13–18	18.1–45	45.1–60	> 60
Fib (g/L)	>2.00	1.50–2.00	1.00–1.49	0.50–0.99	< 0.50
TnI (ng/ml)	≤0.10	0.11–0.59	0.60–1.00	1.01–1.49	≥1.5
AST (U/L)	≤200	201–1000	1001–2000	2001–3000	> 3000
TBIL (μmol/L)	< 20	20–50	51–100	101–150	> 150
Cr (μmol/L)	< 80	80–160	161–250	251–400	> 400
AGI		I		II, III, IV	

*T* Temperature, *GCS* Glasgow Coma Scale, *Lac* Lactate, *PLT* Platelets, *PT* Prothrombin Time, *APTT* Activated part of the Prothrombin Time, *Fib* Fibrinogen, *TnI* Troponin I, *LDH* Lactate dehydrogenase, *ALT* Alanine Transaminase, *AST* Aspartate Transaminase, *TBIL* Total Bilirubin, *Cr* Creatinine, *AGI* Acute Gastrointestinal Injury

### Evaluation effectiveness of EHSS on the prognosis of EHS patients

**Fig. 3:**
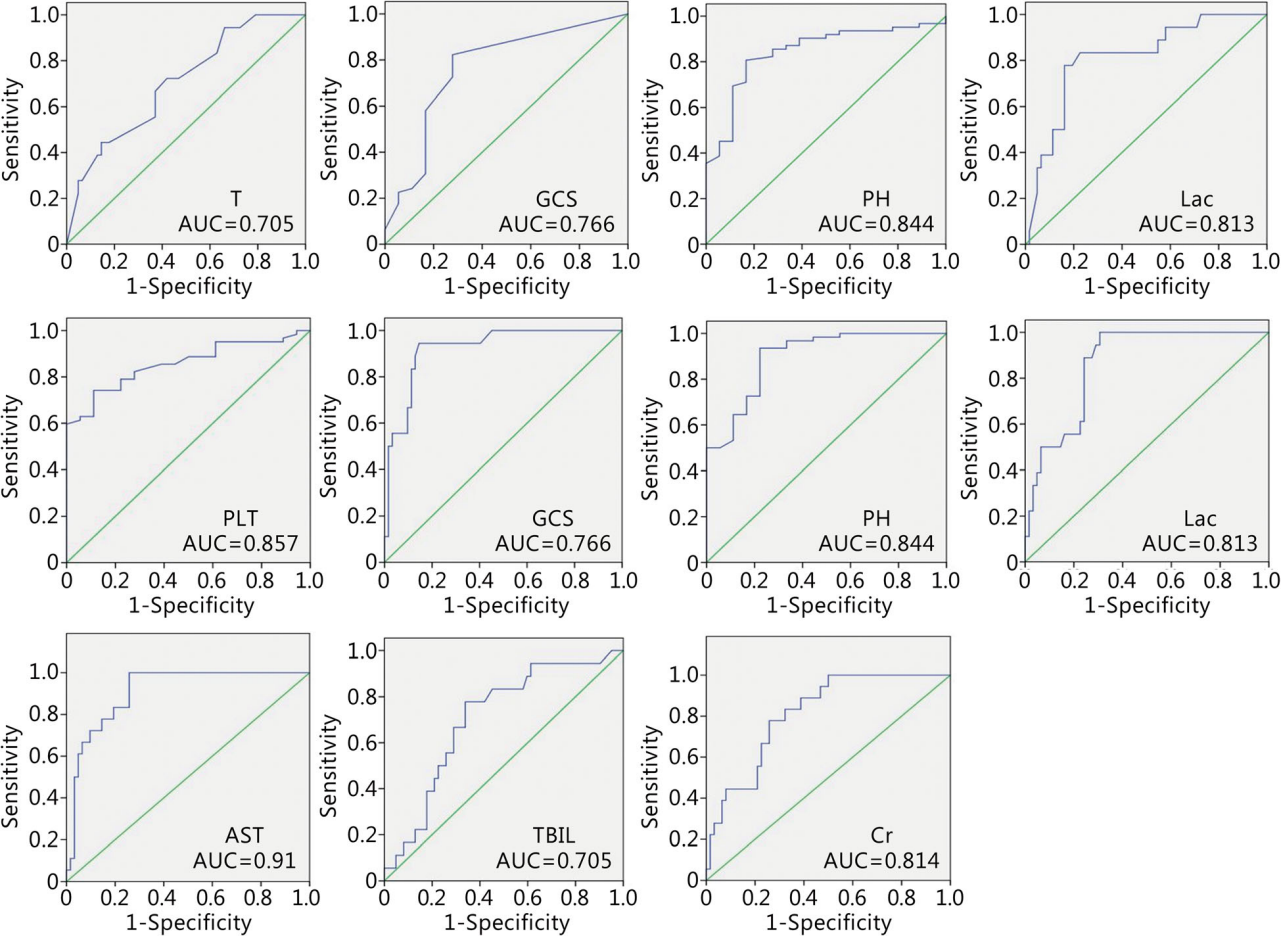
The AUC of each parameter for EHSS was calculated using the EHSS verification database

**Fig. 4:**
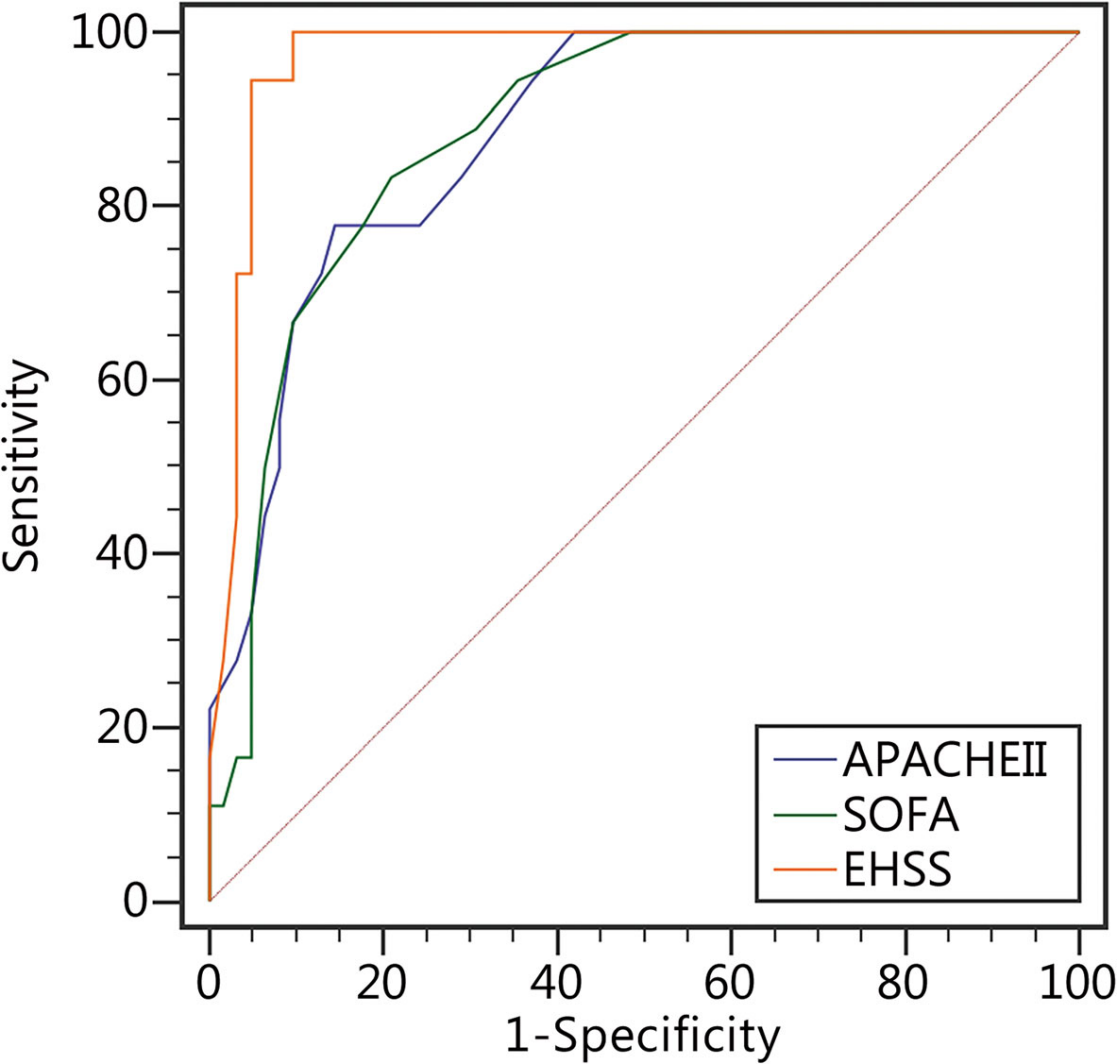
The AUCs of APACHE II, SOFA and EHSS

The AUC of each parameter for EHSS was calculated using the database to evaluate the effectiveness of EHSS (Fig. 3). APACHE II, SOFA and EHSS scores were calculated according to the abnormality levels of the 80 EHS patients; the scores of the survival and death groups from the two scoring systems are shown in Table 7. There was a significant difference in the two scoring systems between the survival and nonsurvival groups. The AUCEHSS was 0.97 (0.905–0.995), the AUCAPACHE II was 0.885 (0.794–0.945), the AUCSOFA was 0.886 (0.795–0.946), (AUCEHSS> AUCSOFA >AUCAPACHE II; AUCAPACHE II compared with AUCSOFA, *P* = 0.9725; AUCAPACHE II compared with AUCEHSS, *P* = 0.0194; AUCSOFA compared with AUCEHSS, *P* = 0.011, Fig. 4). The optimal cut-off point of the EHSS was 22 points; its corresponding sensitivity was 100%, and its specificity was 90.3%. When the EHSS score was no less than 22 points, the mortality rate of EHS patients was as high as 75%.

**Table 7:** The APACHE II, SOFA and EHSS scores of EHS patients in EHSS verification group

Scores	Total (*n* = 80)	Survival group (*n* = 62)	Non-survival group (*n* = 18)	*P*
APACHEII [score, (*x* ± *s*)]	21.2 ± 7.3	18.9 ± 5.9	29.1 ± 6.4	< 0.0001
SOFA [score, M(Q)]	8 (4.3–12.0)	7.0 (4.0–10.0)	13.5 (11.8–15.0)	< 0.0001
EHSS [score, M(Q)]	15.0 (10.0–23.8)	12.0 (8.8–16.2)	29.5 (26.0–32.0)	< 0.0001

*SOFA* Sequential Organ Failure Assessment score, *APACHE II* Acute Physiology and Chronic Health Evaluation II score, *EHSS* Exertional Heat Stroke Score

### Correlation between different EHSS scores and EHS patient prognosis

The mortality rates of EHS patients corresponding to different EHSS score intervals are shown in Table 8. With increasing EHSS score, the mortality rate of EHS patients also increased; however, the mortality rates of the 26–30- and 31–35-point groups showed opposite behaviours. As shown in Table 8, when the EHSS was no larger than 20 points, the mortality rate of EHS patients was 0, and when the EHSS was greater than 35 points, the mortality rate of EHS patients was 100%.

**Table 8:** The mortality at different score intervals of EHSS

Scores	Total(*n*=80)	Survival group(*n*=62)	Non-survival group(*n*=18)	Mortality(%)
0-5	1	1	0	0
6-10	22	22	0	0
11-15	19	19	0	0
16-20	13	13	0	0
21-25	6	4	2	33.3
26-30	9	1	8	88.9
31-35	8	2	6	75
36-40	1	0	1	100
41-47	1	0	1	100

## Discussion

EHS usually occurs in healthy, young people who are engaged in manual labor; even under many interventions, its incidence in athletes and soldiers is still increasing [5]. Therefore, a full understanding of the etiology of EHS and the characteristics of the injuries of various organs, along with a timely and effective evaluation of the severity of the disease in EHS patients, could play an important role in improving the prognosis of patients and reducing the mortality rate, such as taking effective cooling measures or prompting military evacuation from the battlefield.

The scoring system for critically ill patients is an important method to quantitatively evaluate disease severity [25]. The APACHE II scoring system is currently the most widely used and authoritative disease evaluation system for critical illness. This system was designed by the team of Professor Knaus at Washington University in 1985 and is composed of three parts: age, acute physiological score (APS) and chronic health score. The system collects the worst values of various parameters from patients within 24 h after their admission into the ICU to predict the mortality of critically ill patients [13]. The higher the total theoretical score of this scoring system, the more severe the disease it indicates. In general, for common critical illnesses, particularly when the pathological and physiological characteristics of the disease are similar to the inclusion indicators of the APACHE II scoring system, the disease assessment and the prediction of prognosis are often quite accurate [26]. However, studies have continuously confirmed that for some diseases with strong specialist features or some special groups of people, such as pregnant women and nonpregnant women in particular, with characteristic organ injuries or abnormal physiological indicators, all of the above scoring systems have certain flaws. As early as 2006, Professor Stevens et al. [27] used the APACHE III scoring system to retrospectively analyse obstetric patients admitted to the ICU and documented the patients’ demographic characteristics, obstetric and other disease histories and the 20 physiological variables contained in the APACHE III and reached the conclusion that APACHE III scoring had no correlation with the death of obstetric patients in the ICU. Similarly, Ryan et al. [28] conducted a meta-analysis on 25 studies regarding the prediction of the mortality rate of puerperae and critically ill puerperae by APACHE II scoring and found that APACHE II often overestimated the mortality rate of this population. Because EHS has unique pathogenic features of coagulation dysfunction and rhabdomyolysis and the above scoring systems do not include indicators for such injuries, the establishment of a disease evaluation system dedicated to EHS is urgently needed to evaluate this disease severity. When reviewing the establishment process of the previous scoring systems (APACHE II, SAPS II and MODS) for critical illness, it became apparent that their establishment was based on large samples [13, 15, 16]. However, for diseases with strong, specialized features, due to the limitations of the primary disease characteristics and case sources, the sample sizes of the above scoring systems, which were established based on relatively large case sample sizes, could not be reached. For example, the Ranson scale was created based on the data of 100 cases of acute severe pancreatitis by professor Ranson in 1974 [8], and it is still the primary criterion for determining the degree of injury of acute severe pancreatitis. EHS has a sporadic incidence, and it is difficult to obtain large sample sizes of cases. In the present study, a total of 170 cases of EHS were collected in the context of a long-term, multicenter study – one of the largest study sample sizes, either domestic or abroad.

Studies found that the primary thermal cytotoxic effect generated from high heat and the secondary activated systemic inflammatory response were the underlying causes of EHS with the complication of MODS; EHS patients also had manifestations of multiple-organ dysfunction or failure [29, 30]. For any severe disease, its physiological response will be expressed as changes in blood pressure, heart rate, respiratory rate and other basic vital signs [25]. Therefore, in the present study, 42 parameters of body temperature, heart rate, blood pressure, respiratory rate, counts of white blood cells and neutrophils and indicators reflecting injuries to various organs were selected as the screening parameters for the establishment of the EHSS. Because the hospitals in China where the cases were collected were of different levels, during the collection of the cases, we found that some hospitals could not examine some of the parameters listed in Table 1, such as creatine kinase isoenzyme (CK-MB) or brain natriuretic peptide (BNP), which reflect myocardial injury; blood myoglobin (Mb) and urinary myoglobin (uMb), which reflect rhabdomyolysis; fibrinogen degradation products (FDP), which reflect coagulation dysfunction; and C-reactive protein (CRP), interleukin-6 (IL-6) and procalcitonin (PCT), which reflect the inflammatory response. When reviewing the establishment process of the APACHE II, MODS and other authoritative scoring systems for severe diseases [13, 16], without exception, they all noted that the parameters in a scoring system should have the following features: simple, common, easy to obtain and repeatedly detectable. At present, not all hospitals in China can list the eight indicators CK-MB, BNP, Mb, uMb, FDP, CRP, IL-6 and PCT as routine detection indicators. Therefore, the present study ultimately did not list the above eight indicators as the screening parameters for EHSS.

Through univariate analysis and AUC screening, after the initial EHSS parameters were identified, the determination of the final EHSS parameters and the assignment of weights to them were the core issues that needed to be addressed in this study. According to the parameter screening methods of the classical APACHE III, APACHE IV and SAPS II scoring systems for severe diseases, the ultimate determination of the parameters of the scoring system was always obtained from a multivariate logistic regression analysis, and a death risk prediction model was established based on the logistic regression equation. Through statistical analysis, 12 parameters were included in the EHSS to represent the characteristic injuries of EHS patients: high heat, CNS dysfunction and impairments in metabolic, coagulation, cardiac, liver, renal and gastrointestinal functions. These effects are consistent with the characteristics of multiple-organ dysfunction in EHS; therefore, the inclusion of the indicators is appropriate.

The mortality rates corresponding to various assignment intervals of the five parameters T, pH, Lac, AST and TBIL did not show a gradually increasing trend, and the mortality corresponding to the highest score (4 points) assignment interval was instead lower than that of the assignment interval of 3 points (Fig. 2). It is noteworthy that this phenomenon also existed in the establishment process of the MODS scoring system [16]. This behaviour may be due to the small sample size in this study and the uneven distribution of sample size for certain parameters. Existing studies have shown that when the core body temperature exceeded 41.5 °C, the oxidative stress of HS rats was significantly increased, and endotoxin appeared in the portal venous system [31]. When the core body temperature reached 42 °C, HS patients showed systemic endotoxemia [32]. Thus, in the EHSS system established in this study, a body temperature of 41.6 °C–41.9 °C was given 3 points, while a body temperature of no less than 42 °C was given 4 points. For patients with metabolic acidosis, the disease is divided into mild, moderate and severe metabolic acidosis according to the pH value: mild disease had pH values of 7.30–7.36, the moderate disease had pH values of 7.20–7.29, and severe disease had pH values of less than 7.20. In EHSS, we assigned 4 points to pH values of less than 7.20. Numerous studies have shown that Lac values of more than 2 mmol/L are the dividing point of abnormal Lac [33, 34]; when Lac rose every 1.5 mmol/L, the corresponding mortality rate also gradually increased [35]. Studies from Haas et al. [36] showed that when Lac was more than 10.0 mmol/L, the ICU mortality rate of patients was as high as 78.2%, and the hospitalization mortality rate was as high as 78.5%. In this study, when the Lac value was 8.1–10.0 mmol/L, the mortality rate of EHS patients was as high as 76.9%; even in the presence of errors, when the Lac value was more than 10.0 mmol/L, the mortality rate of EHS patients was also as high as 75%. Thus, the distribution of the Lac interval was reasonable in EHSS, which fully reflected the correlation between the Lac level in EHS patients and the prognosis. At present, there is no commonly accepted standard for using the degrees of increase in aminotransferases (ALT, AST) or bilirubin (TBIL, DBIL) to quantitatively evaluate the severity of acute liver injuries; therefore, combining the correlations between the existing data and the mortality rates of EHS patients, we set different assignment intervals for AST and TBIL.

In addition to the above parameters that were correlated with abnormal ICU mortality rates in the 4-point group, we also found that EHSS did not include parameters that reflected rhabdomyolysis. During the process of establishing a scoring model for rhabdomyolysis, McMahon et al. [37] found that creatine kinase (CK) would only affect patient prognosis when the level was more than 40,000 U/L. In the present study, the CK levels were 2852.0 (1441.6-5723.0) U/L in the survival group and 8850.0 (2327.7-35,237.0) U/L in the death group (Table 4). Therefore, while rhabdomyolysis may not be a determinant of the prognosis of EHS patients, its worth still needs to be confirmed by further studies with large sample sizes. Similarly, APTT, an indicator that reflects endogenous coagulation function, was also not included in this scoring system. Bouchama et al. [38] prospectively analysed the blood specimens of 22 HS patients at admission and found that the levels of endothelin, von Willebrand factor (vWF) and intercellular adhesion molecule-1 (ICAM-1) were significantly increased, suggesting that vascular endothelial cells were significantly impaired in patients at the early stage of HS pathogenesis. Injury to vascular endothelial cells mainly activates exogenous coagulation pathways; combined with the results of this study, it can be further speculated that during the early pathogenesis of EHS, exogenous coagulation dysfunction can predict prognosis more accurately. Previous studies showed that troponin I was a good predictor of myocardial injury in HS patients and the severity of HS itself, and when blood TnI was higher than 1.5 ng/ml in HS patients, it indicated the pathogenesis of severe myocardial injury [39]. In the present study, when troponin I was higher than 1.5 ng/ml, the corresponding mortality rate was as high as 75%, consistent with the above results. Because gastrointestinal tract AGI classification is a categorical variable, it could not be screened with AUC. Animal studies confirmed that thermal attack could lead to early necrosis of intestinal epithelial cells [40] and enhanced intestinal permeability [41]; patients would mainly show the symptoms of nausea, vomiting, abdominal pain, diarrhea, watery stool and even gastrointestinal bleeding, which seriously affect patient prognosis [21]. Combined with clinical practices, in the present study, AGI classification, which reflects the degree of gastrointestinal tract injury, was included in the EHSS. At this point, after a series of screenings of parameters, the first EHSS had been established.

In the present study, the APACHE II, SOFA and EHSS scores of 80 EHS patients were calculated, and the sizes of their AUCs were compared. The AUCAPACHE II was 0.885 (0.794–0.945) and the AUCSOFA was 0.886 (0.795–0.946), which had moderate accuracy in determining the prognosis of EHS patients. It is noteworthy that the AUCEHSS was 0.97 (0.905–0.995), indicating that its ability to determine the disease severity of EHS patients was significantly superior to that of the APACHE II and SOFA scores, and the diagnostic results were very good. This result preliminarily confirms that the EHSS, which was established in the first part of this study, has extremely high diagnostic value in judging the disease condition of EHS patients. Moreover, when the EHSS score was no less than 22 points, the risk of EHS patient death started to increase. With increasing EHSS score, the mortality rate of EHS patients also increased. However, the mortality rates of the 26–30- and 31–35-point groups showed opposite behaviours. This discrepancy might be due to the small sample size and uneven sample distribution.

This study still has some limitations. First, this study has a small sample size, which may mean that some parameters that affect the prognosis of EHS patients were not included in the EHSS; therefore, the EHSS still needs to be further confirmed with large samples. The evaluation effectiveness of the EHSS and the mortality rates corresponding to different EHSS score intervals still require further validation in large samples. Second, patients who were in the hospital or ICU for less than 24 h were excluded because we could not obtain the worst values of these patients over 24 h. Some were seemingly serious cases, and some may have been mild. Therefore, we did not see merit in scoring those who had short stays. Excluding patients with comorbidities before they suffered from EHS narrowed the population studied and the potential utility of the EHSS. Third, due to the restriction of sample size, this study could not use a multivariate logistic regression analysis method, meaning that the death risk prediction model could not be established. Fourth, the EHSS has not been verified in different populations, which has certain limitations.

## Conclusions

A total of 12 parameters – T, GCS, pH, Lac, PLT, PT, Fib, TnI, AST, TBIL, Cr and gastrointestinal AGI classification – have been established as EHSS indicators. These parameters have better evaluation effectiveness for the prognosis of EHS patients; low (EHSS< 20) and high (EHSS> 35) scores showed 100% survival and 100% mortality, respectively. EHSS is the first scoring system dedicated to EHS patients, and this study is a pioneering one in the EHS field. The establishment of such a scoring system indicates that the quantification of EHS severity is truly realized, which will have significance in effectively determining disease severity among EHS patients and improving the treatment success rate in the future. However, the EHSS still requires further verification with large samples.

## Data Availability

We agree that the materials described in the manuscript, including all relevant raw data, will be freely available to any scientist wishing to use them for non-commercial purposes, without breaching participant confidentiality.

## Abbreviations

AGI: Acute Gastrointestinal Injury
AKI: Acute Kidney Injury
ALB: Albumin
ALT: Alanine Transaminase
APACHE II: Acute Physiology and Chronic Health Evaluation II
APTT: Activated part of the Prothrombin Time
AST: Aspartate Transaminase
AUC: Areas Under the Receiver Operating Characteristic Curve
BNP: Brain Natriuretic Peptide
BUN: Blood Urea Nitrogen
Mb: Myoglobin
Ca^2+^: Calcium
CHS: Classical Heat Stroke
CK: Creatine Kinase
CK-MB: Creatine Kinase Isoenzymes
Cl^−^: Chlorine
Cr: Creatinine
CRP: C-Reactive Protein
DBIL: Direct Bilirubin
D-D: D-dimer
DIC: Disseminated Intravascular Coagulation
EHS: Exertional Heat Stroke
EHSS: Exertional Heat Stroke Score
FDP: Fibrin degradation products
Fib: Fibrinogen
GCS: Glasgow Coma Scale
Glu: Glucose
Hb: Haemoglobin
HR: Heart Rate
HS: Heat stroke
ICU: Intensive care unit
IL-6: Interleukin-6
K^+^: Potassium
Lac: Lactate
LDH: Lactate dehydrogenase
MAP: Mean Arterial Pressure
MODS: Multiple-organ dysfunction syndrome
N: Neutrophils
Na^+^: Sodium
PCT: Procalcitonin
PLT: Platelets
PT: Prothrombin Time
ROC: Receiver Operating Characteristic Curve
RR: Respiratory Rate
SaO_2_: Oxygen Saturation
SAPS II: Simplified Acute Physiology Score II
SOFA: Sequential Organ Failure Assessment
T: Temperature
TBIL: Total Bilirubin
TnI: Troponin I
TT: Thrombin Time
uMb: uric myoglobin
WBC: White Blood Cell count
