# Correction to: Establishment and effectiveness evaluation of a scoring system for exertional heat stroke by retrospective analysis

**DOI:** 10.1186/s40779-021-00318-3

**Published:** 2021-04-16

**Authors:** Meng-Meng Yang, Lu Wang, Yu Zhang, Rui Yuan, Yan Zhao, Jie Hu, Fei-Hu Zhou, Hong-Jun Kang

**Affiliations:** 1 Department of Critical Care Medicine, The First Medical Centre, Chinese PLA General Hospital, No. 28, Fuxing Road, Haidian District, Beijing, 100853 China; 2 Medical School of Chines, PLA, Beijing, China

**Correction to: Mil Med Res 7, 40 (2020)**

**https://doi.org/10.1186/s40779-020-00269-1**

In the original publication of this article [1], Fig. 2 and Fig. 3 are incorrect, the correct figures are given below. The original publication has been corrected.

**Fig. 2:**
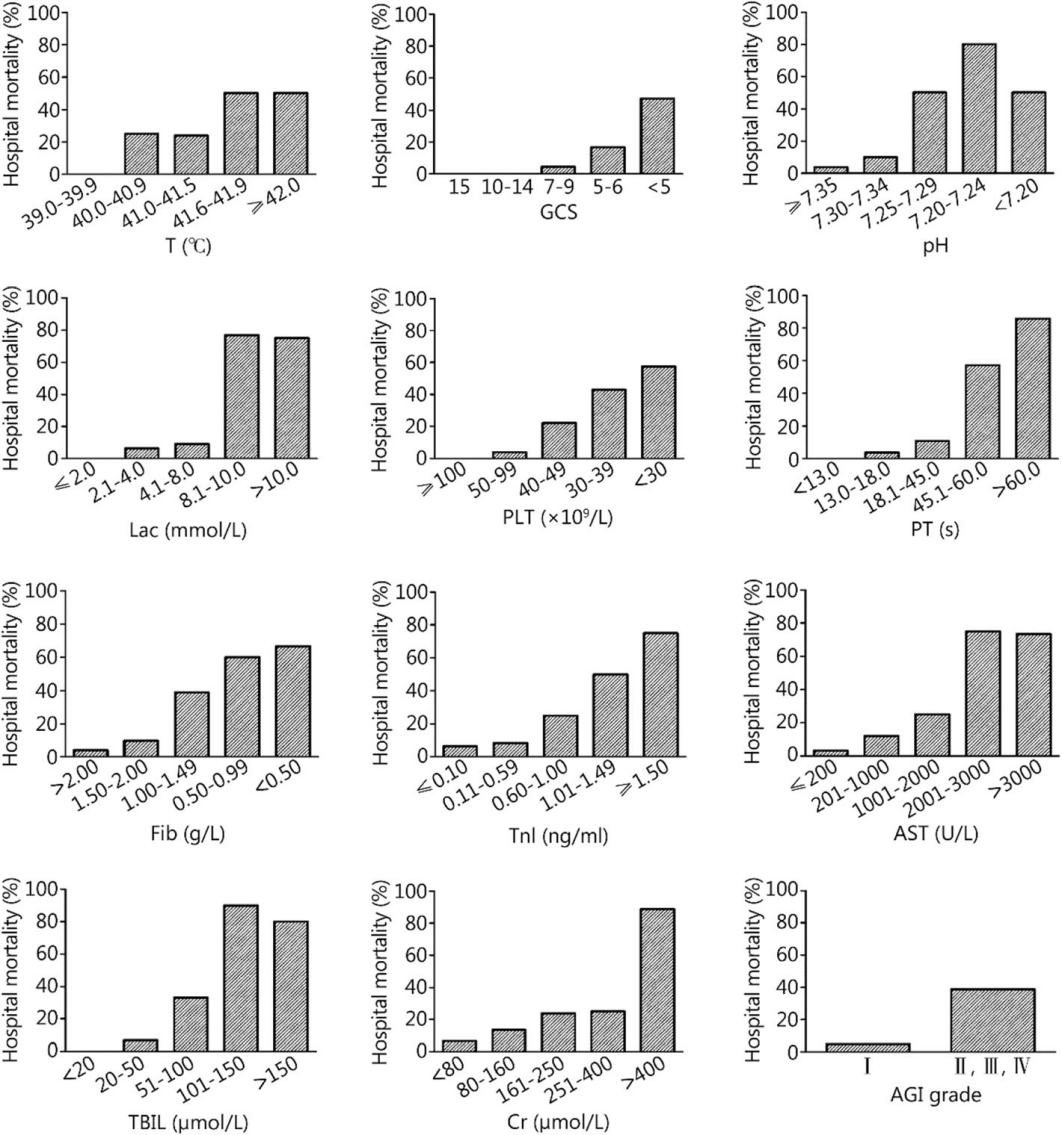
Mortality at different intervals of EHSS parameters

**Fig. 3:**
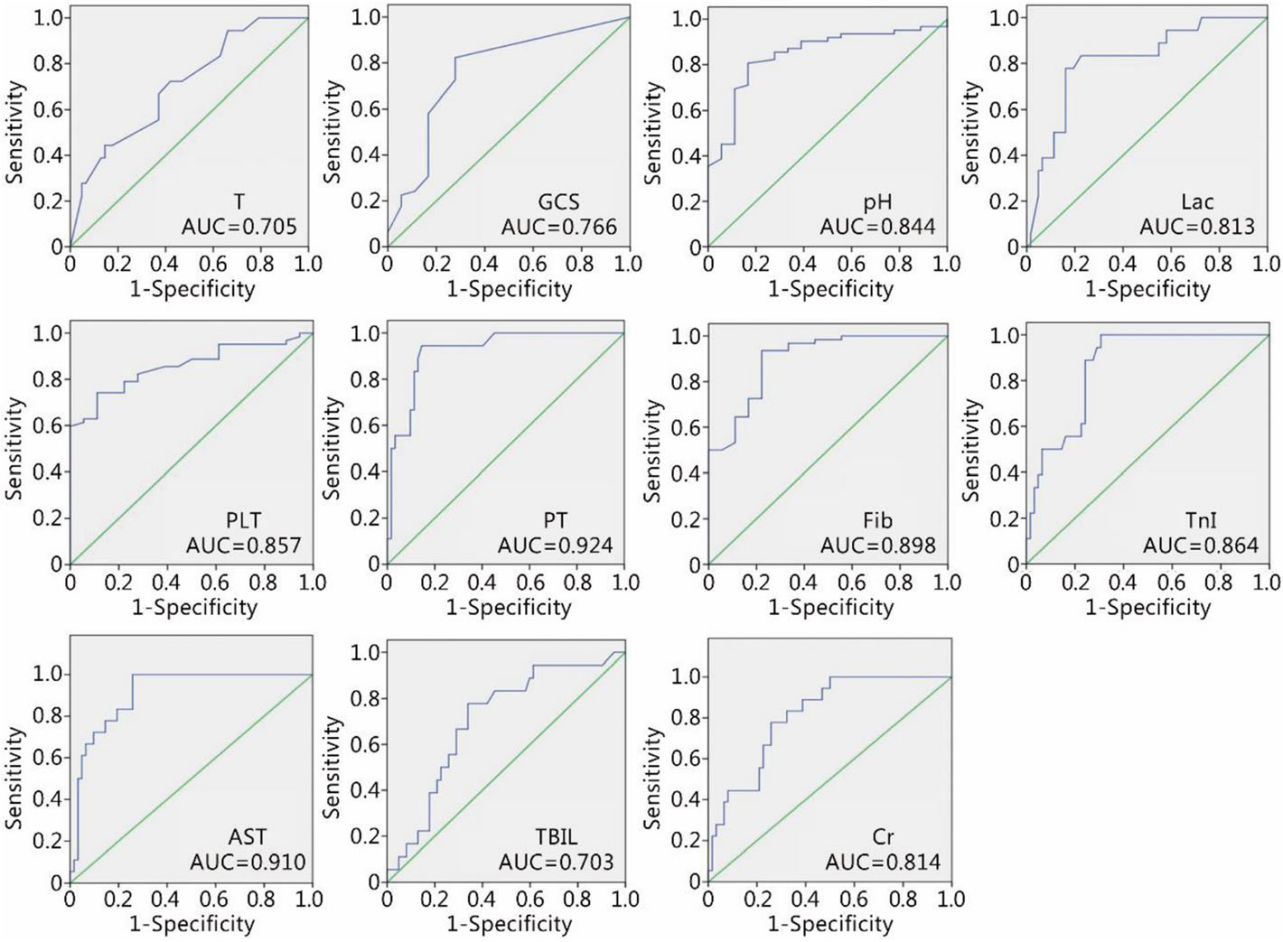
The AUC of each parameter for EHSS was calculated using the EHSS verification database
